# Descriptive ethnobotanical studies are needed for the rescue operation of documenting traditional knowledge

**DOI:** 10.1186/s13002-023-00604-5

**Published:** 2023-09-08

**Authors:** Łukasz Łuczaj

**Affiliations:** Institute of Biology, University of Rzeszów, ul. Zelwerowicza 4/D9, 35-601 Rzeszow, Poland

**Keywords:** Traditional knowledge, Knowledge circulation, Devolution of knowledge, Biocultural diversity, Evolution of science, Theory of science

## Abstract

In this essay, I claim that the primary aim of ethnobiological research is now to document disappearing traditional knowledge. This is an absolute priority due to the rate at which biocultural biodiversity in the world is disappearing. Rather than diverting our efforts into inflating the theoretical part of ethnobotany, we should concentrate on knowledge documentation to facilitate its circulation in the communties that hold it or at least to preserve it for future generations, even in the static form of databases or video recordings.

In a discussion titled “*Should ethnobiology and ethnomedicine more decisively foster hypothesis-driven forefront research able to turn findings into policy and abandon more classical folkloric studies?*” I am tempted to answer no, especially to the second part of the question, concerning folkloristic studies. An explanation follows below.

Vibrans and Casas, in their paper on the evolution of Mexican ethnobotany [1], state that this process is consistent with a model proposed by Schneider [2], based partly on the concept of cyclical advancement of science advanced by Kuhn [3], according to whom the development of any scientific discipline has four main phases or stages. In the first stage, a new frame of reference or language is created. In the second, the main tools are established, allowing some more standardization in research, and the most highly cited papers are published. More intensive collaborations begin. In the third stage, more problems are introduced and combined with the old ones, and more ramifications of the discipline appear. In the fourth stage, general rules are created. At this stage, the discipline becomes the most predictive. Vibrans and Casas state that Mexican ethnobotany and, extrapolating from it, world ethnobotany are between stages three and four.

We do see that ethnobotany has already developed some expectations of how a researcher is to act; its code of ethics; standard ways of gathering data, usually by semi-structured interviews and even some indices allowing quantification of data. Most of these were already laid out in the main handbooks of the discipline [4, 5]. Several theories concerning ethnobotany were also formulated and circulated [6–9]. Yet another attempt of dividing ethnobiology into phases was made by Huhn [10]. The last, fourth stage he distinguished is “indigenous ethnobiology”, science honouring the “folk” perspective, in service of local communities rather than a quantified theoretical science. This approach is very different—rather than seeking “theories”, Huhn emphasizes indigenous perspectives. For indigenous perspectives, the primarily goal now is the preservation of the core of their ethnobotanical traditions from sudden disappearance and helping to provide livelihoods using traditional plant use. Later, Wolverton extended this concept of phases to phase 5 [11], writing extensively about the interdisciplinary nature of ethnobiology (and ethnobotany within it) and its goal in taking part in solving environmental problems. He writes: “I argue that ethnobiology is preadapted to be a scholarly umbrella for a number of disciplines that concern human–environment interactions, suggesting that one goal of Ethnobiology 5 is to bridge traditional academic boundaries in order to broaden the community of ethnobiologists. Another goal of Ethnobiology 5 is to capitalize on and communicate the relevance of ethnobiological scholarship for solving problems related to contemporary environmental and cultural crises”. Recently phase 6, the decolonization of ethnobiology was proposed [12]. In this essay, I will endeavour to explain why the documentation of traditions and descriptive ethnobotany are so important for humanity, regardless of which phase they belong to: the indigenous stage 4 or the decolonization stage 6.

Ethnobotany is often placed at the edge of botany and cultural anthropology. The first ethnobotanical works were of a descriptive character, where the use of plants was described, and the plants’ scientific names were identified, not always in a rigorous fashion. In this way, useful plants of many indigenous people in North America were recorded before this knowledge was completely lost. Similar efforts were made in other parts of the world. Some traditional knowledge (TK) is encoded in botanical literature, e.g. old herbal manuscripts and printed works, especially in China and Europe, but also in Mexico and a few other countries and regions [13–15].

The Earth is undergoing dramatic changes: urbanization, modernization, migration, destruction of habitats and climate change cause the loss of ethnobotanical traditional knowledge along with the loss of local languages and dialects, resulting in the “devolution” of this knowledge [16]. Most of TK is transmitted orally. The work of an ethnobiologist is like rescuing shipwrecked people. Humanity has recorded a good share of TK but most of it is getting lost. The disappearance of human cultural heritage is progressing, and we should focus more efforts on organizing efficient data documentation, facilitate intergenerational transfer and activate local communities to do it for themselves, especially in the form of incorporating traditional knowledge into educational curricula. This cultural destruction is happening along with the disappearance of languages and natural habitats [17, 18]. Although many languages and dialects are dying out, some of them have been brought back from the brink of extinction. Similarly, although plant use traditions are dying, they can still be recorded from its last holders. In the region of Sardinia, the making of acorn flour was still practised only by one old lady [19]. This shows the great importance of the proper archiving of traditional knowledge as in future once archaic foods, e.g. acorn products, may re-enter the economy [19, 20].

Even in better studied regions, a large part of TK remains undocumented. The level to which it remains under-recorded is much larger than the distribution of plants and their plant associations. Let us take the example of Europe. Several European countries have very detailed floristic atlases but corresponding atlas maps concerning ethnobotanical phenomena were only published in Poland [21]. No comparable work is available for other European countries, though large ethnographic archives concerning plants can be found in some countries, e.g. Estonia [22]. Now, a large programme of documenting traditional ethnobotanical knowledge has been carried out in Spain [23], and many local ethnobotanical works are being performed throughout Europe, but in some countries, this journey is just starting. Moreover, old folkloristic publications often only cover selected regions, being absent from others.

We can imagine that in countries with a multi-ethnic population that uses a variety of languages and with mainly an oral tradition, the volume of traditional knowledge to be stored and preserved is many times larger. Thus, it is surprising that there are relatively few scientific journals focused on ethnobotany, little money is spent on documentation, and descriptive ethnobotanical monographs are so scarce. It is sad that so often descriptive papers are rejected only on the grounds of their descriptiveness. There are still many countries for which a search using the keyword “ethnobotany”, and the name of the country brings no more than a few dozen references. Practically, I think that wherever there are gaps in the documentation of not only wild food plant or medicines but sometimes more specialized categories specific to a given community, such as fish poison, dye plants and the like, knowledge is usually lost quicker [24]. Again, it is enough to illustrate this gap searching databases using the terms “plants” and a given country or ethnic group. In contrast with this, most countries have their botanical floras, sometimes in many volumes, and extensive literature on local vegetation.

While understanding that ethnobotany is on the edge of botany and cultural anthropology, we should emphasize its rootedness in history and economy. Good (hi)stories do not necessarily require hypotheses; they are good pieces of history in and of themselves. As far as economic aspects are concerned, both in the field of getting to know the chemical composition of useful plants, as well as that of bringing them to broader cultivation, we are only at the beginning of the journey.

One of the most unusual examples of historical ethnobotanical works is an article on a specimen of royal fern worshipped for centuries in a churchyard in Sweden [25]. This historical perspective and singularity of one plant or one person go against the trend of interviewing as many informants as possible at any price. On the other hand, some papers presented in international journals now contain too few interviews, and not because there were no more informants to be found but because of the hurry in which studies are made and published. We should explore ethnobotany in its whole scope of methodology—from qualitative in-depth small-scale studies, to large-scale quantitative works. Ethnobotanical discourse is now often based on building data matrices: respondent × species × use type, which are later analysed in a quantitative way. However, most of this data had been gathered during interactions with real people, often voice- or sometimes even video-recorded. What is missing for me in modern ethnobotany are the voices, the anecdotes, all the stuff which is discarded when making the final raw matrix to be deposited in a repository as a spreadsheet, or not even; to be turned into a few tables. This is often left unpublished due to the sheer lack of time. There is a big gap between short and rigorous dry scientific reports on one side of the spectrum and popular guidebooks of useful plants with no reference to the sources on another—we have to fill in the middle with well-documented local descriptive ethnobotanical studies, e.g. available as illustrated books written in local languages. In their book on ethnoecology of a Hungarian-speaking region in Romanian Carpathians, Babai et al. [26] placed portraits of all their informants at the end of the book. This acknowledges the importance and authorship rights of the local TK holders. Also, the practice of including word by word accounts of informants was once widespread in ethnobotany (see, for example the description of bracken rhizome processing by the indigenous Americans in Boa’s works [27]) and has now been replaced by tables and data matrices, continuing to be practised only in ethnolinguistic studies or purely anthropological studies. This kind of knowledge documentation took into account the linguistic intricacy of the knowledge usually lost in turning the field material into data matrices. Unfortunately, even humanities journals tend to coerce authors to increase the use of quantitative methods [28].

While seeking new interesting and useful theories and paradigms in ethnobotany, I believe that we should not obsessively strive for quantification at any price, anywhere, but only where it is obviously needed. Unfortunately, many ethnobotanical papers use numbers and indices only to look scientific, and lack not only soul but beauty and basic raw material such as full transcripts of interviews or stories, photos or videos. They only present numbers and indices, and even the lists of species are provided in “additional material”, which may be lost with time. The indices they are decorated with are like Christmas decorations on a simple and beautiful conifer tree of classic descreptive ethnography. It is more and more difficult to perform ethnobotany as the science of discovery, as Richard Evans Schultes did, but it is still possible to make it thoroughly, allowing the reader to access as much raw material as possible, for example documenting knowledge using videos, sound recordings and by making it available to the local communities and other researchers (bearing in mind the ethical issues associated and the danger of authorities, and especially regimes, using this information against local communities). Deloria, already in 1969, claimed that too much anthropological work was made with little connection to what should be studied for the good of indigenous communities. He argued that such works create a whole network of scientific studies showing a certain intellectual construct of an ethnic group with little connection with reality, serving mainly the intellectual circles of anthropologists themselves [29].

Contrary to the advocates of the quantification of ethnobotany mentioned in the beginning of my essay, I am afraid ethnobotany may be reaching its limits in the search of grand theories but has a great future as an applied science, in seeking out useful species and activities to preserve cultural heritage and biodiversity as well as in increasing people’s livelihoods. We need basic ethnographic research even to understand contemporary capitalism as so many value chains start with remote communities gathering economically important species for the global economy. As Tsing put it “[t]o understand capitalism (and not just its alternatives)… we can’t stay inside the logics of capitalists; we need an ethnographic eye to see the economic diversity through which accumulation is possible” [30]. In another passage, she says “to learn anything we must revitalize arts of noticing and include ethnography and natural history”.

If a rigid approach is needed in ethnobotany, I see it rather in the proper identification of specimens (e.g. using barcoding), phytochemical investigations or in creating large, well-constructed and open-access databases (the Native American Ethnobotany website [31] is a good example); or extensive regional or tribal monographs of traditional knowledge. However, I am against placing too many standardized methodological restraints on ethnobotany. Science is a social phenomenon, and ethnobotany is an interdisciplinary domain deeply rooted in the humanities. However fascinated, we may be by Popper’s falsificationism of hypotheses or *P* values, the greatest discoveries in science are often made due to mistakes, play, intuition, etc. Paul Feyerabend in his anarchist theory of science contained in his book “Against Method” [32] praises a kind of social science which is messy, chaotic and pluralist, which breaks stereotypes and makes new discoveries in unexpected ways. But if quantitative ethnobotany becomes a paradigm, a *sine qua non* to defend a thesis or get published, I am against it. Let us keep ethnobiology as pluralist as possible. I am not talking about making “bad” science but about exploring the whole spectrum of unusual methods, approaches and insights. Recently, Leonti and colleagues [9] suggested that all major discoveries in ethnobotany have been made on the basis of qualitative (primary data) studies, not quantitative (statistical) studies, which confirms Feyerabend’s point.

To illustrate how ethnobiology gets disconnected from life, here are two examples from a recent conference I attended. One young scholar made their PhD barcoding a rare kind of traditional wild food. They tested many samples containing several related species. I was so excited after the talk that I came up and asked which of the samples tasted the best. Unfortunately, the student answered that they had not eaten this food, in spite of working on it in the laboratory for a few years. In the same conference, another presenter also showed us an overview of the use of a few related wild food species. Again, I asked the same question. And the answer was the same: “I have never eaten it”. If ethnobiology is going to become just database exploration, I refuse to take part in it.

A few decades ago, some cultural anthropologists attempted to be more connected with ecology and the material part of culture, trying to tie cultural phenomena with ecological reality and constraints. Here, we can mention the works of such scholars as Julian Stewart or Marvin Harris [33, 34]. In a similar way, we can look at contemporary scholars attempting to apply or create scientific theories in ethnobiology ([7] and see discussion: [8, 9]), many of them taken from ecology. I myself am tempted to do so and have applied the classic island biogeography theory in ethnobiology [31, 35], given that making theories and testing them is an important and natural part of scientific thinking. In spite of this, I strongly believe that the main agenda of ethnobiology now is knowledge documentation, application and dissemination, however imperfect it may be. We have to bear in mind that TK is not static and is sometimes difficult to express in the written form or even as video recordings. Still, a written record is better than nothing and can be helpful in restoration projects. A quantified hypothesis-driven approach in ethnobotany can only be one part of this fascinating interdisciplinary domain. Traditional knowledge has intrinsic value, as does biodiversity, and can be explored and described without hypotheses, but preserving basic reasonable scientific rigour using proper species identification, repeatable phytochemical methods and simple descriptive statistics.

## Data Availability

Not applicable.

## Abbreviation

TK: Traditional knowledge
